# A Rare Case of Mixed Adenoneuroendocrine Carcinoma of the Ileocecal Valve

**DOI:** 10.7759/cureus.3942

**Published:** 2019-01-23

**Authors:** Hector H Gonzalez, Jamie L Skrove, Radhika Sharma, Javier Sobrado

**Affiliations:** 1 Internal Medicine, Florida Atlantic University Charles E. Schmidt College of Medicine, Boca Raton, USA; 2 Internal Medicine, Larkin Community Hospital, South Miami, USA; 3 Osteopathy, Larkin Community Hospital, South Miami, USA

**Keywords:** ileocecal valve, neuroendocrine tumor, mixed adeno-neuroendocrine tumor, crohns disease, mixed adenoneuroendocrine tumor

## Abstract

Mixed adenoneuroendocrine carcinoma (MANEC) is an uncommon neoplasm with uncertain pathophysiology. In order to be classified as MANEC, the tumor must contain at least 30% neuroendocrine cells and 30% adenocarcinoma. The standardization of MANEC treatment has historically been difficult due to the lack of diagnostic histological classification. In 2010, the World Health Organization (WHO) finally recognized this uncommon condition as a specific colon cancer entity in the hopes of better specifying treatment options in the future.

We present a case of high-grade MANEC of the cecum with metastasis in 3/10 lymph nodes to further characterize the diagnostic modalities and treatment options of the disease. MANECs only account for 3%-9.6% of all colorectal cancers and only eight cases have been reported in the cecum to date, making the following case report exceptionally rare.

## Introduction

A mixed adenoneuroendocrine carcinoma (MANEC) is an uncommon neoplasm of the gastrointestinal tract that must consist of least 30% neuroendocrine cells and 30% adenocarcinoma to be classified as such [1]. The initial description of MANEC dates back to a report published by Cordier in 1924 [2-4]. Since then, not only have there been several cases reported inconsistently under a variety of naming systems, but the classification criteria have also changed, making it historically difficult to adequately characterize the disease [2]. More recently, in 2010, the World Health Organization (WHO) finally recognized this uncommon, yet highly malignant condition as a distinct colon cancer entity, ultimately moving from the previous classification of mixed exocrine-neuroendocrine carcinomas to the current, mixed adenoneuroendocrine carcinomas (MANECs).

MANECs only account for 3%-9.6% of all colorectal cancers but have also been reported in several organ systems beyond the gastrointestinal tract, including the gallbladder, pancreas, and uterine cervix [1,4]. The diagnosis of MANEC is based on tumor architecture and is achieved by immunohistological identification, as imaging features are often nonspecific and carcinoembryonic antigen (CEA), carbohydrate antigen (CA) 19-9, and CA 125 levels are often normal [1].

With only eight cases of MANEC reported in the cecum to date; in this paper, we present yet another rare case of a high-grade mixed adenocarcinoma/neuroendocrine tumor of the cecum with metastasis in 3/10 lymph nodes.

## Case presentation

**Figure 1:**
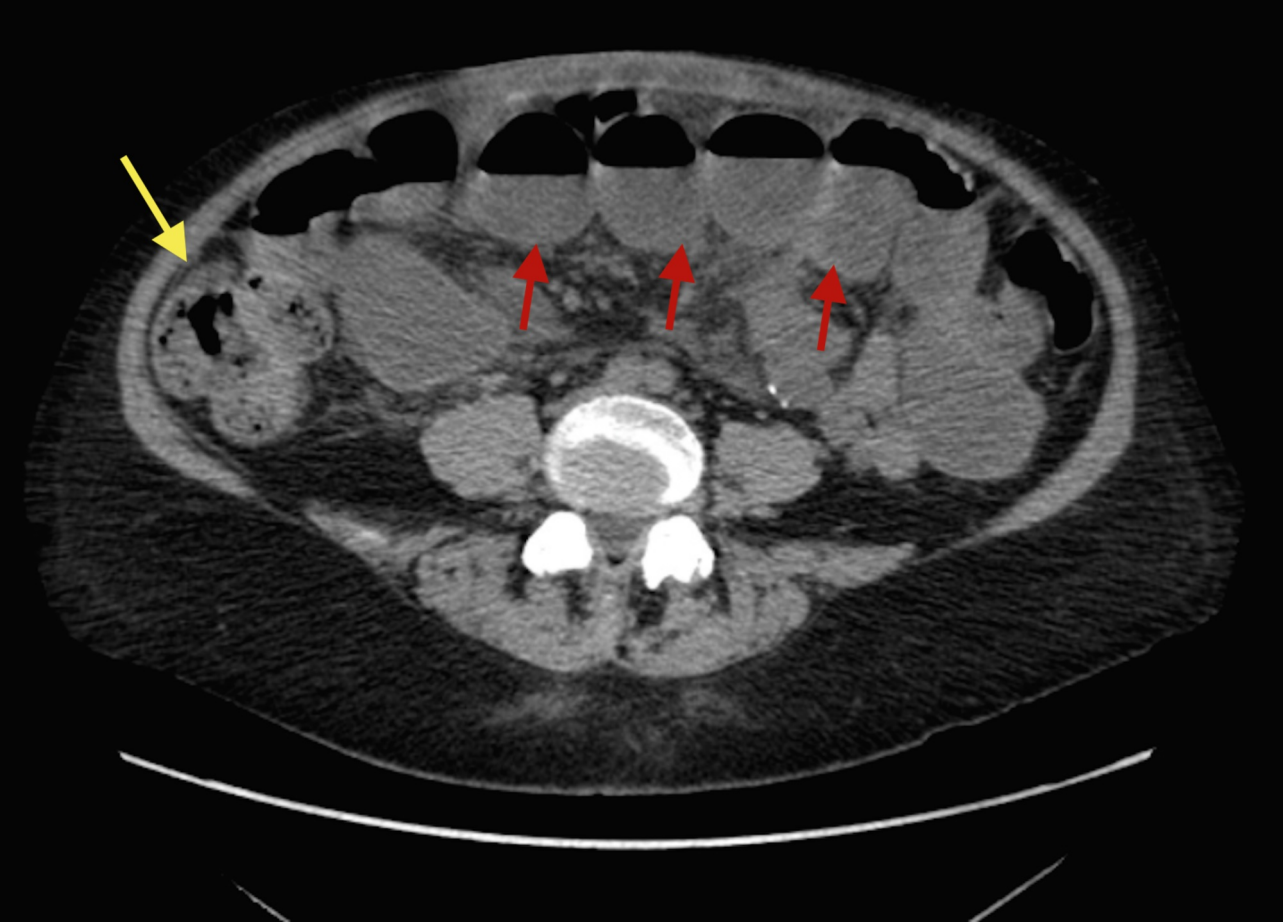
Axial computerized tomography scan of the lower abdomen showing high-grade partial versus complete distal small bowel obstruction (yellow arrow), with fluid-filled dilated small bowel loops (red arrows).

A 56-year-old female with a past medical history of Crohn's disease presented to the emergency room (ER) with complaints of diffuse abdominal pain, nausea, and vomiting. Her physical exam was remarkable only for diffuse tenderness to palpation of the abdomen and mild to moderate distention. Computed tomography (CT) abdomen/pelvis showed high-grade partial vs complete distal small bowel obstruction with terminal ileum thickening (Figure 1).

**Figure 2:**
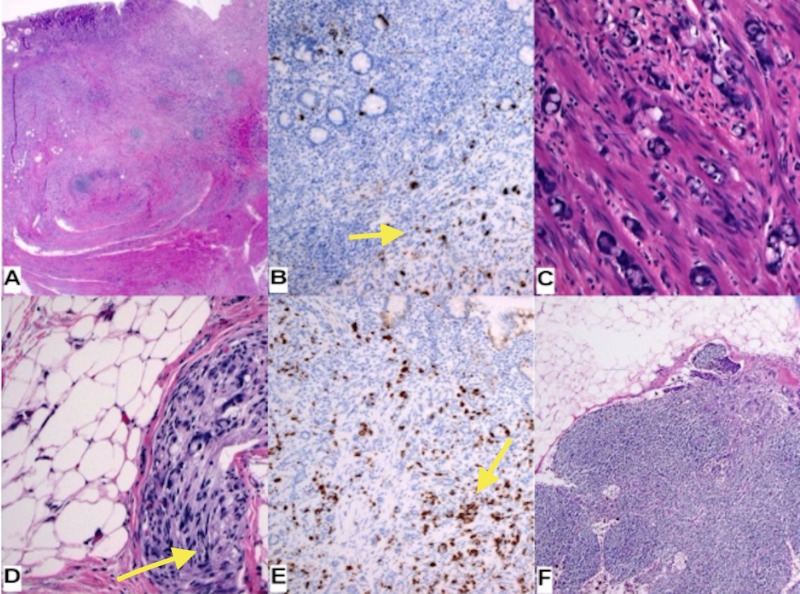
A. Haemotoxylin and Eosin stain (HE) 1.25x B. Chromogranin A 10x - focal positive (yellow arrow) C. HE 20x D. HE perineural invasion (yellow arrow) E. Synaptophysin 10x - focal positive (yellow arrow) F. HE lymph node metastasis, keratin AE1-3 positive

CEA and CA19-9 were within normal limits. Colonoscopy resulted in ileocecal valve thickening suspicious for carcinoma. Surgery was performed and the specimen pathology showed a high-grade mixed adenocarcinoma/neuroendocrine tumor with metastasis in 3/10 lymph nodes. Immunohistochemistry was focally positive for chromogranin and synaptophysin (Figure 2).

Post-surgery, positron emission tomography-computed tomography (PET-CT) was performed and showed changes from the recent ileocolonic resection with reanastomosis. There was no definitive evidence of metastatic disease.

## Discussion

The first description of MANEC dates back to a report published by Cordier in 1924. Since then, there have been several cases inconsistently reported under a variety of names, which historically made it difficult for adequate characterization of the disease. In 1987, Lewin introduced yet another inadequate tri categorical naming system for such neoplasms: collision tumors, combined tumors, and amphicrine tumors, creating even further confusion [2]. In 2010, the World Health Organization (WHO) finally recognized this rare and highly malignant condition as a distinct colon cancer entity, ultimately moving from the previous classification of mixed exocrine-neuroendocrine carcinomas to the current, mixed adenoneuroendocrine carcinomas (MANEC). The presence of neuroendocrine cells in non-endocrine neoplasms and vice versa is a common phenomenon and has been documented frequently, and so there is a wide spectrum of combinations of neuroendocrine and exocrine tumors with various degrees of differentiation of both components [2]. MANECs are histologically defined as carcinomas composed of both gland-forming epithelial and neuroendocrine neoplasms and must contain at least 30% of both to be classified as such. These types of mixed tumors are extremely rare, and they are notoriously aggressive with a high metastatic potential, which appears to arise from the neuroendocrine component of the tumor [5].

For further classification of these neoplasms, MANEC was divided into high, intermediate, and low-grade tumors. High-grade MANECs are more aggressive tumors, corresponding with a high Ki-67 proliferation index (60&-90%) and a worse prognosis [2,4]. They are composed of an adenomatous or carcinomatous component along with a poorly differentiated (small, intermediate, or large cell type) neuroendocrine component. High-grade types with a small or large cell neuroendocrine component are rare. They appear as polypoid masses or ulcerating lesions, most similar histologically to their small or large cell NEC counterparts of the lung [2,4]. Immunohistochemically, the small and large cell components must stain positively for two of the following for diagnosis: chromogranin, synaptophysin, and/or CD56 [1-2]. The non-neuroendocrine component of high-grade MANEC may be composed of tubulovillous or villous adenoma, adenocarcinoma, or squamous cell carcinoma [2]. High-grade neoplasms have been documented in the esophagus, stomach, large bowel, and in the anorectal segment. The prognosis of high-grade lesions depends on the stage and tumor type with a worse survival associated with distant metastases [5].

Intermediate grade MANECs are composed of two subtypes: mixed adenocarcinoma-neuroendocrine tumor and amphicrine carcinoma. The first subtype is composed of degrees of differentiation of the exocrine component - tubular, papillary, or mucinous adenocarcinoma and well-differentiated neuroendocrine cells [2]. As opposed to the high-grade neoplasms, the exocrine component of the mixed adenocarcinoma-neuroendocrine tumor is more aggressive than the neuroendocrine counterpart [2,6]. This subtype has been documented in the esophagus, stomach, ampulla of Vater, ileum, and colon with a higher prevalence in males. The second subtype, amphicrine carcinoma, is a tumor in which both exocrine and neuroendocrine features are co-expressed by the same neoplastic cells [2]. This subtype tends to be extremely rare, with only four reports of occurrence in the stomach.

Low-grade MANECs are classified as mixed adenocarcinoma-neuroendocrine tumors (MANETs) and are composed of well-differentiated exocrine and neuroendocrine cells. The exocrine component is tubular or villous with low- or high-grade dysplasia and the neuroendocrine component is carcinoid. Low-grade MANECs are typically indolent and have an excellent prognosis, as there has been no evidence of tumor recurrence in the documented cases to date [2].

Generally, MANECs only account for 3%-9.6% of all colorectal cancers but have also been reported in several organ systems beyond the gastrointestinal tract, including the gallbladder, pancreas, and uterine cervix [4]. Because these tumors present with non-specific symptoms, they are usually an incidental finding found during exploratory procedures [4]. Diagnosis is done mainly on a histopathologic basis, as imaging features tend to be nonspecific and CEA, CA19-9, and CA125 levels are often normal [6]. Because diagnosis is revealed only with the development of metastases, it is imperative that immunohistological identification be prompt and accurate [4]. High-grade MANECs must stain positive for two of the following for diagnosis: chromogranin, synaptophysin, and/or CD56 [2]. The neuroendocrine portion of this tumor does not, however, behave normally in the immunoreactive sense with the positivity of the three tests above at 60%-70%, 75%-90%, and 50%, respectively [1]. The proportion of CD133 positivity may also aid in the diagnosis of these rare tumors, to determine whether the neuroendocrine component is indeed neuroendocrine versus a dedifferentiated area of adenocarcinoma [1].

MANECs in the cecum are extremely rare, as only eight cases have been reported to date. All previous cases of cecum MANEC were reported in women, arising in about the sixth decade of life [1]. Due to the rarity of this neoplasm, the epidemiology has been extrapolated to previously reported cases. There is a greater predisposition in male/female, with a 1.5:1 ratio [1]. A review of the literature has shown several cases of MANEC arising in the setting of long-standing inflammatory bowel disease. The inherent increased risk of malignancy in long-standing inflammatory bowel disease has been well-documented. The proposed pathophysiology involves multipotent stem cells with bidirectional differentiation [1]. However, the exact mechanism of development of this neoplasm in the setting of inflammatory bowel disease is not clear. In our case, the neuroendocrine component was positive for synaptophysin and chromogranin. It was also positive for Keratin AE1-3, as lymph node metastases were noted. Due to the rarity of these neoplasms, treatment for aggressive MANECs remains inconsistent. When considering the management of the localized disease, the only curative approach is one that involves complete surgical resection of the primary tumor and metastases [7]. Due to the lack of published studies, recommendations that have been applied to neuroendocrine carcinomas and adenocarcinomas have been used to build the current management regimen of MANECs [8]. Surgical resection is recommended for neuroendocrine tumors >2 cm in diameter that have either invaded the muscularis propria or with lymphovascular invasion/locoregional lymph node involvement [8]. Surgical resection, along with lymphadenectomy, has also been recommended for colorectal adenocarcinomas as well [8]. In patients for whom initial surgical resection of distant metastases is not possible, conversion therapy should be considered post-chemotherapy. Although MANECs contain combined histology, previous studies indicate that distant metastases are usually composed of one component; a 5-fluorouracil-based regimen has been shown to be effective for metastatic colorectal adenocarcinomas and a cis-diamminedichloroplatinum-based regimen for neuroendocrine carcinomas [8].

It is also imperative to address the more aggressive histological component of the neoplasm with adjuvant chemoradiotherapy. A neoplasm composed of a well-differentiated neuroendocrine component should be treated as adenocarcinoma and a MANEC with a poorly differentiated neuroendocrine component as a neuroendocarcinoma [2]. The aggressive nature of the neuroendocrine component is based on the mitotic index and the Ki-67 proliferation index [6]. A primarily platinum-oriented adjuvant treatment is used in the treatment of MANEC due to its effectiveness in the treatment of small cell lung carcinoma, a neoplasm that shows histological similarities to that of the small cell neuroendocrine component type [8]. Previous studies have shown a combination of cisplatin and etoposide to be associated with a 67% response rate and a median survival of 19 months in patients with metastatic anaplastic neuroendocrine carcinomas of the colon and rectum and a median progression-free survival of 8.9 months in patients with colorectal neuroendocrine carcinomas [5,8]. The use of a FOLFOX (FOL – folinic acid F – fluorouracil OX – oxaliplatin) regimen, a vincristine-based regimen with adriamycin, and a regimen based on carboplatin have also been reported to show improved survival [8]. If the metastatic lesion has a predominantly adenocarcinoma component, the use of a combined 5-fluorouracil and oxaliplatin-based regimen has been shown to increase progression-free survival when compared to 5-fluorouracil therapy alone [8]. Previous studies also show that adjuvant chemoradiotherapy, when given preoperatively, has improved survival in advanced rectal adenocarcinoma as well as provided an increased benefit of sphincter preservation [8]. Although its efficacy remains uncertain when given preoperatively for neuroendocrine tumors, it may be a promising avenue for its impact on survival. It is clear that additional comparative diagnostic and treatment studies are needed in the future to further build guidelines for MANEC [9].

## Conclusions

Mixed adenoneuroendocrine carcinoma (MANEC) is a rare neoplasm with uncertain pathophysiology. Due to the non-specific symptoms, highly aggressive nature, and metastatic potential of these tumors, it is vital that physicians remain cognizant of MANECs for their differential diagnoses. It is also clear that additional comparative, diagnostic, and treatment studies are needed in the future to develop proper therapeutic management of this complex condition.
